# The use of plant-derived exosome-like nanoparticles as a delivery system of CRISPR/Cas9-based therapeutics for editing long non-coding RNAs in cancer colon cells

**DOI:** 10.3389/fonc.2023.1194350

**Published:** 2023-06-14

**Authors:** Tatiana Hillman

**Affiliations:** Biotechnology, LAL4Bsynbiotics L.L.C, Los Angeles, CA, United States

**Keywords:** CRISPR, exosomes, nanocarrier, colon cancer, gene-based therapeutics

## Abstract

Colon cancer is one of the leading causes of cancer in the United States. Colon cancer develops from the many gene mutations found in the genomes of colon cancer cells. Long non-coding RNAs (lncRNAs) can cause the development and progression of many cancers, including colon cancer. LncRNAs have been and could be corrected through the gene-editing technology of the clustered repeats of the clustered regularly interspaced short palindromic repeats (CRISPR)-associated nuclease 9 (CRISPR/Cas9) system to reduce the proliferation of cancer cells in the colon. However, many current delivery systems for transporting CRISPR/Cas9-based therapeutics *in vivo* need more safety and efficiency. CRISPR/Cas9-based therapeutics require a safe and effective delivery system to more directly and specifically target cancer cells present in the colon. This review will present pertinent evidence for the increased efficiency and safety of using plant-derived exosome-like nanoparticles as nanocarriers for delivering CRISPR/Cas9-based therapeutics to target colon cancer cells directly.

## Introduction

Colon cancer is a common gastrointestinal cancer in the world. In the United States, colon cancer frequently occurs more than breast, lung, and prostate cancers. Only lung cancer precedes colon cancer as the first leading cause of death. In the U.S., about 1.5 million individuals have colorectal cancer (CRC). Colonoscopies have decreased the rate of CRC. As a result of improved treatments, such as chemotherapy, immunotherapy, and colectomy, the 5-year survival rate of CRC patients is nearly 64% (1). The distribution of microorganisms and their metabolites are related to colon carcinogenesis; however, the CRC mechanisms of progression are still not as precise (2). Noncoding RNAs (ncRNAs) can cause the development of colon cancer. ncRNAs are part of a class of non-protein coding RNAs that do not become translated into proteins but can affect cellular processes. Non-coding RNAs internally residing in exosomes may be involved in the process of tumorigenesis and development, in which ncRNAs may also aid in significant intracellular communication within a tumor (3). Long non-coding RNAs (LncRNAs) have a DNA sequence length of 200 nucleotides and affect many biological processes, such as cell proliferation, differentiation, development, apoptosis, and metastasis (4).

LncRNAs bind to RNA, protein, and DNA, forming RNA-RNA, RNA-DNA, and RNA-protein structural complexes, which can lead to the control of gene expression through diverse mechanisms. These diverse mechanisms can consist of transcription, mRNA stability, and translation (5, 6). There is evidence emerging for lncRNAs being especially vital for colon cancer development and progression (7, 8). A study identified 200 lncRNAs expressed in colon tumors when analyzing data from RNA sequences in the TCGA dataset (9). LncRNAs also affect the prognoses of patients, the proliferation of cells, cell apoptosis, metastasis and invasion, the cycle of cellular division, epithelial-mesenchymal transition, drug resistance, and cancer stem cells. The expression of lncRNAs is associated with the pathogenicity of colon cancer (10). For example, the lncRNA zinc finger E-box binding homeobox 1 with antisense 1 expression is increasingly amplified in colon cancer tissues when compared to healthy colonocytes (11).

Although many improved cancer therapies exist, such as chemotherapy, targeted biological therapy, radiation therapy, and combination therapies, there is still a high propensity for relapse with increased resistance to chemo-radiation therapy. There are also many toxic side effects that occur from each therapy applied (12). Consequently, new and novel therapeutic strategies for cancer treatments are currently needed. The clustered regularly interspaced short palindromic repeats (CRISPR)-associated nuclease 9 (CRISPR/Cas9) system has provided an additional effective therapy for cancer. Presently, CRISPR/Cas9 is a type of molecular scissor with application in many studies, such as cancer research, the discovery of therapeutic drugs, treating cognitive illnesses, and being applied to plants. CRISPR is contained within the adaptive immune system of prokaryotes to provide immunity against viruses by cleaving foreign viral DNA (13, 14). Cancer is a genetic illness with multiple DNA and RNA mutations present in cellular genomes (15, 16).

These genetic mutations in the cancer cell genome can be corrected to overcome cancer (17). Much scientific evidence supports that CRISPR/Cas9 can correct cancer-causing genetic mutations (18). Essential data and evidence confirm the potential of the CRISPR/Cas9 system to target the protein-coding genome and emend lncRNAs present in humans (18–20). A few diseases have the potential to rapidly become treated with CRISPR-Cas9 technology *via* an *ex vivo* approach; however, for CRISPR-Cas9 therapeutics to achieve clinical success, the system must be directly applied to patients. A direct administration of CRISPR-Cas9 therapeutic requires a safe and precise delivery of its systems *in vivo*; however, these delivery aspects of CRISPR-Cas9 therapeutics are not wholly developed (21). Plant-derived exosome-like nanoparticles (PENs) may offer a promising delivery system for administering CRISPR-Cas9 therapeutics and treating colon cancer. PENs can potentially serve as effective nanocarriers of CRISPR-Cas9 therapeutics designed to target lncRNAs in human colon cancer cells through an oral administration. According to Kim et al. (22) Plant-derived exosome-like nanoparticles (PENs) are predicted and expected to develop into effective therapeutic techniques for treating diseases or delivering drugs. This study’s main focus and purpose was to describe PENs as effective delivery vehicles for drugs and biomolecules into the colon for treatment of colorectal cancer (CRC). This review will provide a brief background of the CRISPR-Cas9 structure and function, a survey of the current challenges of existing CRISPR-Cas delivery technology, and present supporting evidence for using PENs as efficient nanocarriers for CRISPR/Cas9-based therapeutics *via* an oral administration into the lower digestive tract.

## Survey methodology

### Structure and features of the CRISPR/Cas9 system

**Figure 1:**
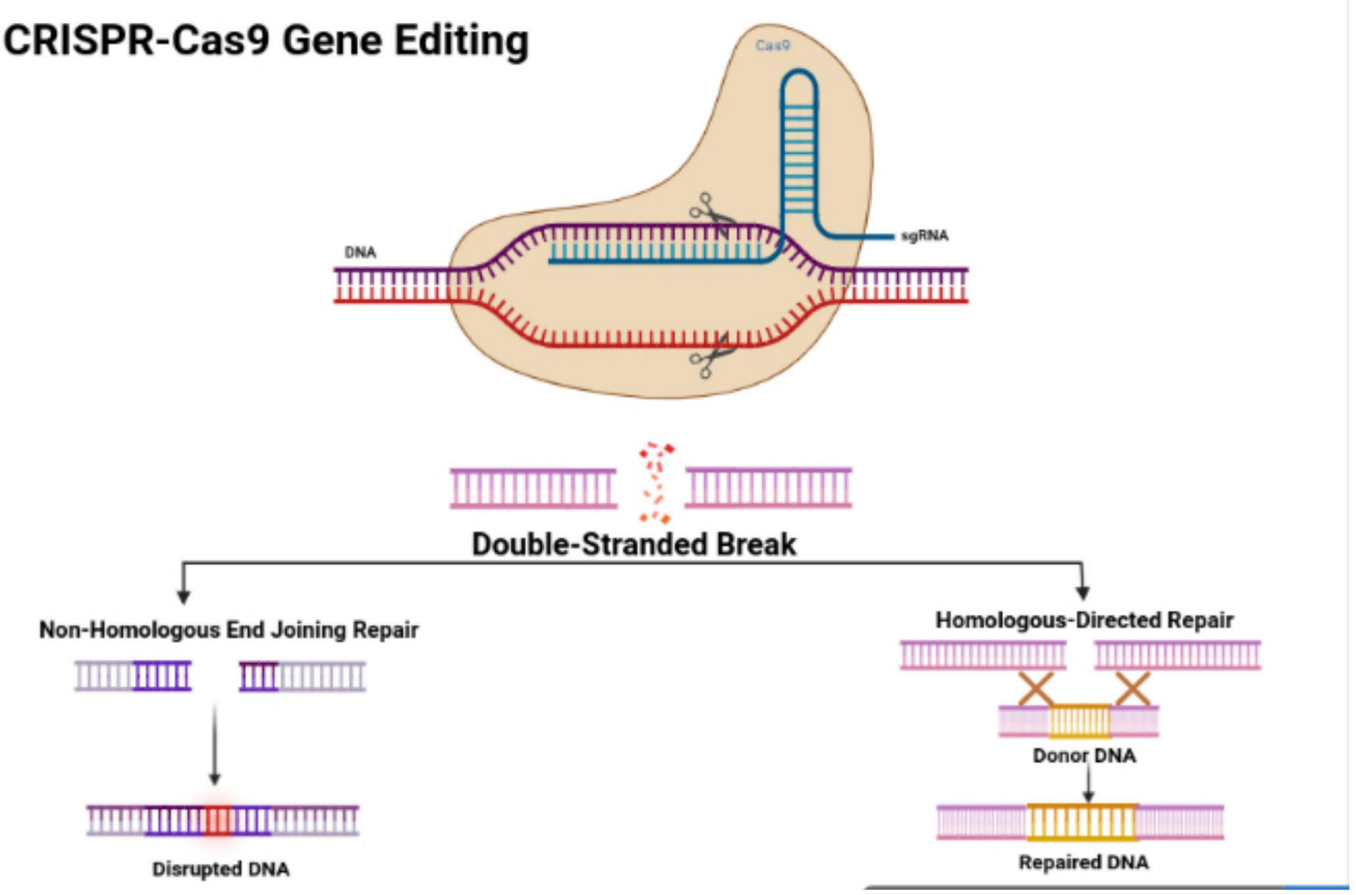
CRISPR/Cas9 structure and features. Figure displays the structure of the CRISPR-Cas9 protein with its complex containing the sgRNA bound to the target gene. The figure also shows the process of non-homologous end joining repair (NHEJ) and homologous directed (HDR). The figure was retrieved from Boti et al. (31, 32).

In 1987, Ishino and his colleagues first found CRISPR in *Escherichia coli*. They discovered that the clustered repeats had a series of spacer-type sequences, later called CRISPR. Since then, many researchers have found more varieties of CRISPR/Cas systems. There are three categories, including type I, II, and III, and many diverse subtypes divided based on their differing mechanisms (23, 24). The CRISPR/Cas9 system is a type II CRISPR system found in *Streptococcus pyogenes*. It is the most widely applied system in mammals since it is highly efficient and accurate. CRISPR/Cas9 is the first engineered CRISPR/Cas system for editing genomes since it contains single guide RNAs (sgRNA) that can be easily programmed with a recognition sequence of a short 20 nucleotides in length (13, 25, 26). The sgRNA is composed of CRISPR RNA (crRNA), which consists of a sequence complementary to the targeted site and a transactivating crRNA (tracrRNA), which is partially complementary to the crRNA (27–29). The Cas9 nuclease is also a component of the CRISPR/Cas9 system, in which the RNAs guide the Cas9 protein to the targeted sites while also activating the Cas9 nuclease activity. The sgRNA couples with the Cas9 protein, forming a more combined complex and recognizing the targeted site, which is a complementary DNA sequence flanked at the 3’ end and adjacent to the protospacer adjacent motif (PAM) (30) (**Figure 1**). The PAM primarily consists of NGG or NAG, in which N can be either A, T, G, or C, and the PAM assists with initiating the DNA double-stranded breaks (33).

### CRISPR and colon cancer

LncRNAs regulate cell proliferation, and LncRNA dysregulation controls the proliferation of colon cancer cells. The overexpression of ZEB1-AS1 modulates cell growth by increasing p21-activated kinases 2 (PAK2) expression by absorbing miR-455-3p into cancerous colon cells. LINC01082 becomes downregulated in cancerous colon tissues with the upregulation of LINC01082, causing the inhibition of cell proliferation in SW480 colon adenocarcinoma cells. Inhibiting gene expression reduced cell proliferation by suppressing LINC01296 gene expression in SW480 and SW620 colon cancer cells (31).

By silencing LINC01082, cell proliferation was inhibited by targeting the miR-21a of colon cancer cells (34). Increased taurine-upregulated gene 1 (TUG1) expression has been confirmed in the colon cancer tissues, with p63 downregulated, increasing TUG1 expression in HCT116 and LOVO cancerous colonocytes. Knocking down TUG1 expression inhibits the cell proliferation of HCT116 and LoVo cells (35).

**Figure 2:**
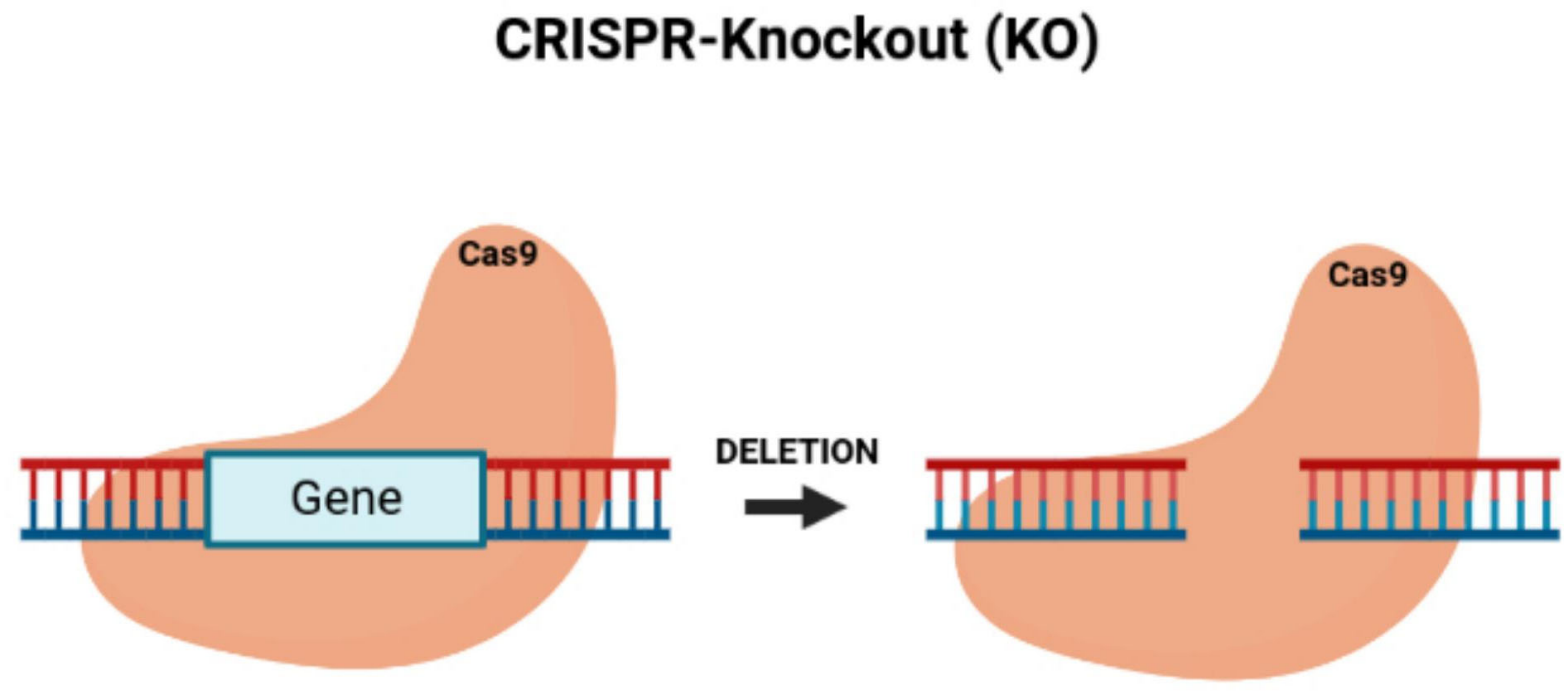
CRISPR/Cas9-knockout of targeted oncogenes. Figure shows the CRISPR/Cas9 deletion of a targeted gene, which represents an oncogene.

By knocking down TUG1, the proliferation of colon cancer cells can be blocked while also reducing tumor growth *in vivo* (36). Since the dysregulation of cell growth causes the development of tumors by activating proto-oncogenes and inactivating tumor-suppressor genes (37), CRISPR/Cas9 is an effective genome engineering technology that offers new benefits for treating cancer with the potential to modify multiple genes. The CRISPR/Cas9 system can inhibit tumor growth by its knock-out of oncogenes (38) (**Figure 2**). Li et al. (39) applied CRISPR/Cas9 with the single-guide RNA (sgRNA) to build gene-modifying tools to edit mutations in the beta-catenin genes (40). The beta-catenin gene mutations are carcinogenic, in which correcting these genes can reduce their mutation rate, offering a new cancer gene therapy (41). Zhang et al. built HSV oncolytic viruses using the CRISPR/Cas9 system, and they genetically altered the HSV-1 genome to treat colon cancer (42). The secretory mucin termed MUC5AC causes the development of colon cancer due to drug resistance. Pothuraju et al. performed a gene knockout of MUC5AC for treating colorectal cancer cells *via* RNA interference and CRISPR/Cas9 modification *in vitro* and mouse models *in vivo* (43). Chakraborty D et al. discovered that targeting NPY/Y2R can treat colon cancer and help monitor angiogenesis. Using CRISPR/Cas9 gene modification, a knockout of the VEGF-A gene inhibited angiogenesis in mice treated with a Y2R antagonist (44). ELAVL1 is an RNA-binding protein of HuR that improves the expression of HuR. Using CRISPR/Cas9 technology to knockout HuR increased the activity of apoptosis. HuR can be a suitable therapeutic target for colon cancer (45). Takei et al. found that ERO1α had increased expression in colorectal cancer, causing a poor prognosis and increasing the development of colorectal cancer. ERO1α was knocked out, and the growth of the colon cancer was inhibited, with the cell proliferation and metastases being decreased with the reduced expression of the integrin-beta1 on the cells’ surface (46).

Pyruvate carboxylase (PC) increases the development and metastasis of colon cancer, causing a lesser time of survival with a poorer prognosis. A knockout of PC using CRISPR technology blocked and reduced tumor growth (47). Oh et al. confirmed that tubulin acetyltransferase αTAT1 controls Wnt1 expression to induce microtubule acetylation and enhances malignant tumor growth and invasion. CRISPR/Cas9 technology was used to knock out αTAT1, decreased tumor invasion, and inhibit colon cancer development (48). Membrane-associated loop CH protein 2 (MARCH 2) regulates autophagy and the transport of vesicles. Xia et al. discovered that MARCH 2 was increasingly expressed in colon cancer with a poorer prognosis and lower survival rate (49). MARCH 2 was knocked out, and the endoplasmic reticulum stress was stimulated with the inhibition of cancer cell growth and induction of apoptosis (50). Chen et al. confirmed that FAPP2 controls the Wnt/β-catenin signaling pathway and enhances the growth of cells of a tumor. FAPP2 is increasingly expressed in colon cancer cells and increases the expression of colon cancer cells. FAPP2 was knocked by CRISPR/Cas9 technology, the cell growth of tumors was blocked, and the tumorigenicity was decreased (51). CLCA1 was knocked out using the CRISPR system (49). After knocking out CLCA1, the proliferation, and migration of colon cancer tumor cells were increased, and the high level of expression of CLCA1 caused the inhibition of the Wnt signaling transduction and reduced the epithelial-mesenchymal-transition (EMT) process that blocked tumor growth (52).

Presently, researchers are investigating CRISPR-Cas9 gene-based therapeutics’ efficacy as a treatment for cancers of the lung, head, breast, liver, and of colon (53). ncRNAs, consisting of microRNAs, lncRNAs, and circularRNAs, all play a vital role in the development and progression of cancer. Approximately 99 percent of the human genome contains ncRNA regions with limited protein-coding activity (30). Therefore, CRISPR/Cas9 can target these non-coding regions and may become an efficient strategy for gene-based cancer therapy (30).

### Current CRISPR delivery mechanisms

However, treating cancer will require the CRISPR/Cas9 components to interact directly with target cells by traversing multiple physical barriers (54, 55). Additionally, for the gene editing system to operate accurately, the Cas9 protein and the sgRNA must successfully enter the nucleus simultaneously at the same time (54). As a consequence, the type of delivery system is significant for applying gene editing therapies *via* the CRISPR/Cas9 technology. Microinjections, hydrodynamics, and electroporation delivered CRISPR/Cas9 components *in vitro*, yet these delivery modes are less effective *in vivo*. The use of viral vectors is also lacking in their capability to enter into the clinical use of therapeutics because viral vectors produce an immunogenic response, have increased off-target effect, are expensive, and have a smaller capacity for loading therapeutics (56). Viral vectors are most widely and commonly used for CRISPR/Cas9 delivery (49). However, adenoviral vectors cannot encapsulate the 10,000 base pairs of a CRISPR/Cas9 DNA plasmid vector. Using non-viral vectors, such as lipid nanoparticle carriers, can fully encapsulate the extensive and lengthy CRISPR/Cas9 DNA plasmids (49). For these reasons, applying non-viral vectors has created a new research field in which non-viral vectors are more advantageous than viral vectors. Non-viral vector systems include lipid nanoparticles and gold (Au) nanoparticles (49).

Non-viral vectors, through nanotechnology, can provide nanocarriers, such as polymers, lipids, and metal-organic structural complexes, to package cancer therapeutics, which have a low rate of causing an immune response, have sufficient delivery of cargo capabilities and have high biological compatibility (57, 58). Specifically, lipid-based delivery systems can transport through challenging physiological obstacles, are easy to synthesize, are frugal to compose, and can be mass-produced (57). When divided into different categories, lipid nanoparticles depend on their formation method and physicochemical properties, including solid lipid nanoparticles, nanostructured lipid carriers, niosomes, and liposomes. Solid lipid nanoparticles (SLNs) are formed from solid lipids (59), have a spherical shape, and have sizes of 50 and 1000 nm. Cancer therapeutics can be encapsulated in SLNs by encapsulation in the lipid matrix *via* a solid solution, a lipid shell model, and a drug-enriched shell (60). The solid solution model distributes the anticancer therapeutics into the lipid matrix. While forming the drug-enriched shell, the shaping of the shell occurs during the cooling of the SLNs in the phase separation of the drugs from the lipids. The drugs precipitate in the lipid shell model before the recrystallization of the lipid. As a result, SLNs can carry hydrophilic or hydrophobic drugs and more accurately deliver anticancer drugs.

### Nanotechnology-based delivery systems for colon cancer

Through a ligand-receptor binding mechanism, ligand-altered nanoparticles can accumulate in a tumor. These types of nanoparticles are termed active targeting nanoparticles (61). Through passive targeting, these ligand-modified nanoparticles can gather in the tumor and then transit into tumor cells *via* active targeting, causing more specific and improved therapeutic effects. For the last three years, this active targeting strategy for nano-drug targeted delivery systems for colorectal cancer has implemented the receptor-ligand binding strategy, which included many increasingly expressed CRC receptors, such as a folate receptor, CD44, epithelial cell adhesion molecule (EpCAM), CD133, nucleolin, EGFR, αvβ3 integrin receptor, MUC1, P-selectin, SSTRs, glucocorticoid receptor, sigma-2 receptors, CXCR4+, checkpoint kinase 2, lipoprotein receptor-related protein-1, mannose receptor, carcinoembryonic antigen, hyaluronic acid receptor, and N-acetyl-D-glucosamine. A few nanoparticles can provide an accurately targeted therapy for CRC patients during stimulation conditions, such as external magnetic fields, reactive oxygen species (ROS), near-infrared (NR), and temperature. In recent years, much research has emphasized the usefulness of magnetic hyperthermia, in which magnetic nanoparticles are injected into a tumor during stimulation from external magnetic fields and then by giving patients a local radiofrequency with hyperthermia (62). During a research study, superparamagnetic chitosan-based nanocomposites were used to deliver SN-38 through a combination with hydrophilic polymeric prodrug poly (L-glutamic acid)-SN-38, which displayed an optimized aggregation in CRC and created a more facile internalization into cells with the aid of a topical magnetic field. Additionally, complexes of magnetic nanoparticles acquired a tumor inhibition ratio of 81% in a mice model with CRC xenografts (63). Dabaghi et al. confirmed that by combining magnetic hyperthermia with magnetic nanoparticles carrying the cancer drug, 5-FU which was more impactful for treating CRC and, more preferably, therapeutic in the mice model of CRC. The magnetic nano complex system can amplify tumor-targeted aggregation and enhance the inhibition of colorectal cancer progression. Because tumors produce excess amounts of ROS to transition the tumor from a state of inflammation into cancer, Zhang et al. formed a ROS-sensitive and hydrogen peroxide-eliminating matter through a cycle polysaccharide, then applied it to manufacture functional nanoparticles to carry the irinotecan as cargo. When a high concentration of ROS in a cancerous colon is stimulated, the irinotecan is released. An oral administration of the nanoparticles was given, and then the tumorigenesis with the development of colitis-induced CRC mice was inhibited.

NIR can be applied to function as an external stimulus to activate the drug release from nanoparticles at the target site. Yadav et al. (64) formulated a compacted shell-crosslinked micelle to carry and deliver indocyanine green (ICG) and doxorubicin (DOX), which could release the drugs when responding to NIR stimulation. When stimulated by NIR, the ICG produced ROS that fractured the micelle structure by degrading the disulfide bond in the micelles, and then an enlarged quantity of DOX would be released. Anugrah et al. (65) formed a hydrogel of alginate to encapsulate ICG and DOC, in which the diselenide bonds could be damaged by the ICG generation of the ROS and then release the DOX by a gel-sol transformation when stimulated by NIR light. Thus, NIR light-responsive drug delivery can be used as a targeted therapy for CRC. However, the immune system will identify and consume many nanomaterials after an intravenous and systemic administration that will cause negative side effects and decrease the therapeutic effects. Additionally, because CRC causes poor-quality vascularization, the number of nanoparticles injected intravenously can be reduced in amount before arriving at the colon with cancer, limiting the effectiveness of the nanoparticles (66). Consequently, most researchers are developing nanomaterials to be orally administered that can maintain a stable targeting of CRC. An oral administration can also augment compliance from patients.

A pH-dependent system can be used since the gastrointestinal tract is organized into the gastro, small intestines, and intestinal crissum, which differ in pH at each part of the GI tract. The stomach’s pH is approximately 1 to 3, the pH of the small intestines increases to 5.5 to 6.8, and then the pH of the colon is around 6 to 8 (67). Researchers were inspired by the enteric coating of tablets, and they initiated the development of a nano-drug delivery system consisting of enteric-coated materials to create pH-sensitive drugs to be released in the colon (68). When the pH values are low, the nanoparticles will remain complete and unbroken, but in high pH values, the dissolution of the coating materials would cause the nanoparticles to expand and attach to the colon, releasing the drug into the targeted area of interest. Eudragit and polysaccharides are used the most as coating materials.

Pectin can be extracted from polysaccharides to provide protection from the acidic gastrointestinal environment (69). Mohamed et al. (70) coated SLNs with pectin and dry skim milk, which released the curcumin cargo in the colon. As a result, the oral administration of curcumin was significantly increased and improved. An additional research study applied beta-lactoglobulin to carry irinotecan and prevent its destruction in the stomach, in which the drug was released in the small intestines. An MTT assay showed that these nanocarriers are more toxic to HT-29 cancer cell lines and AGS than a free drug. As more enteric-coated materials are found, the targeted nano-drug delivery systems established upon pH levels will be used more in targeted therapies for CRC. However, a pH-dependent system has limitations, such as the differing GI tracts between different individuals. Taymouri et al. (71) formulated a polymeric-coated capsule to carry and deliver simvastatin (SIM) into the colon, which was responsive to pH and time. To increase the solubility of the drug, a researcher used anti-solvent crystallization methods to form the nanosuspension of SIM and examined whether the nanosuspension could generate an improved anticancer effect for HT-29 versus the free drug. Subsequently, a capsule consisting of ethyl cellulose and Eudragit S100 was formulated, in which the ethyl cellulose produced a controlled release with a more time-dependent release, and the Eudragit had a solubility that was pH-dependent. This nanosuspension was infused with sodium dodecyl sulfate for freeze-drying to become loaded into the capsule. Their results confirmed that the SIM was not released into the stomach but into the colon. SIM loaded into nanoparticles increased cytotoxicity to HT-29 compared to free SIM. pH- or time-dependent nano-drug delivery systems have some limitations, such as the pH-dependent nanoparticles may not completely target the colon since the colon has a pH of 6.8, which is a similar pH present in the small intestines of ph 7.4, and the time is not fully certain for a gastrointestinal transit of the nanomaterials, which compels the time-dependent nanoparticles to sometimes neglect the targets (72). Designing nanoparticles to release drugs dependent on the degradation by microbes in the colon is an additional strategy for targeted CRC therapy. Because there are 400 classifications of microbial flora in the colon, including Escherichia coli and Clostridium (73), a few polysaccharides can only be broken down into smaller monosaccharides by the anaerobic microbiota of the colon and then implemented by the bacteria as their source of energy. However, these polysaccharides can become metabolized or consumed by gastric and intestinal enzymes (74). Therefore, polysaccharides are immensely resourceful in the enzyme-targeted therapies of CRC. Polysaccharides can regulate the location of the drug release and are biodegradable with much biocompatibility as natural polymers.

A study formulated a colonic enzyme-responsive dextran-based oligoester crosslinked with nanoparticles to deliver 5-FU. The nanoparticles released 75% of the 5-FU *in vitro* within 12 hours of its incubation with glucanase with no drug release under the pH-like conditions of the stomach and the small intestines (75). Tiryaki et al. (72) constructed nanoparticles to contain organic and inorganic materials, which included silica aerogels coated with dextran and dextran aldehyde. After coating with dextran and dextran aldehyde, the encapsulated drugs in silica aerogel particles became released in the colon as the dextran was degraded by the dextranase.

Dos Santos et al. (76) made chitosan nanoparticles and loaded them with 5-FU, in which the nanoparticles were encapsulated with microparticles formed from decomposed starch and pectin. Retrograde starch can prevent degradation by enzymes of the upper digestive tract, and their results confirmed that fewer nanoparticles were released from the microparticles than from the nanoparticles in the gastrointestinal lumen. Therefore, polysaccharides are central to enzyme-responsive colon-targeted materials and preparations. Researchers have designed nanoparticles with many different targeting mechanisms and functions. Rajpoot and Jain (77) framed dual-targeted nanoparticles that consisted of folate-modified SLNs and enteric polymer-coated alginate microspheres that encapsulated the nanoparticles. Using the pH-sensitive enteric polymer allowed the enteric-coated microbeads to release the drug in the colon after an oral administration. Combining folate with the nanoparticles led to the nanoparticles targeting CRC. Another study used near-infrared, which could be tracked, persistent luminescence mesoporous silica nanoparticles coated with lactobacillus reuteri biofilm (LRM) to prevent the drug from digesting and transiting completely into the colon. The LRM lengthened the time of the release of the 5-FU and protected the 5-FU from digestion in the stomach to impel the nanoparticles for actively targeting the colon since the LRM could identify the biological components of CRC, including adhesin (78).

Drug delivery systems can be designed based on many different environmental signals. Ma et al. (79) designed a pH- and enzyme-dependent nanocarrier to carry and deliver chemotherapeutic drugs. Eudragit RS nanoparticles were used to encapsulate indomethacin, 5-FU, and curcumin, respectively, through nanoprecipitation and then inserted into biphasic microcapsules consisting of chitosan and hydroxypropyl methylcellulose by use of aerosolization. Coating the microcapsules with enteric Eudragit S100 could shield the microcapsules from degradation in the stomach. The microcapsules were released, and then their release would end when reaching the colon because chitosan was metabolized and consumed by bacterial enzymes, causing the drugs to accumulate in CRC. Since nanoparticles are highly effective in animal models with CRC, clinical applications with nanoparticles hold much promise and potential. However, there are still many limitations of nano-drug delivery systems. The main obstacle is producing nano-materials and formulations on a large industrial scale and examining their safety and efficacy in preclinical trials.

The compositions of nanomaterials for targeted CRC therapies are more complex, making the preparation process of these nano-materials more challenging to synthesize and replicate. Because the physical and chemical components of platforms should be regulated during the production process, manufacturing methods are increasingly made more difficult with a high cost of production. Through microfluidics, Valencia et al. (80) formed a self-assembly lipid-polymer and lipid-quantum dots (QDs), which formed stable and uniformly structured nanoparticles. However, this complex composite of nanoparticles causes much potential toxicity to patients. Therefore, it is required to choose a model that reflects the development of human CRC to examine the possibility of toxicity during preclinical studies (81). During clinical trials, it was discovered that nanomaterials tend to decrease the toxicity of drugs and not enhance the drugs’ efficacy (82). Many scholars have investigated and found that most nanoparticles compile at tumors utilizing the EPR effect, including after utilizing actively targeted nano preparations, but this EPR effect is more frequent in animals as CRC patients have different EPR effects, which impact the effectiveness of nano-preparations (83, 84). Thus, a more personalized treatment is essential that can use nanoparticles specific for patients with an intense EPR effect to improve efficacy (73). Preparations could be used to amass the tumor separate from the EPR effect, such as utilizing temperature-sensitive-based hydrogel for more localized and targeted treatment of CRC.

The CRISPR plasmid, mRNA, and gRNA of CRISPR-Cas9 technology that is negatively charged can be encapsulated in positively charged lipid nanoparticles during an electrostatic interaction (38). The lipid nanoparticles used as nanocarriers aid the CRISPR/Cas9 in being transported through the cell membrane, but they also protectively shield the CRISPR/Cas9 components from further degradation and immune response. Lipids that are more commercialized are solid and effective vehicles of delivery (38). Lipofectamine and RNAiMAX are formed to deliver many different CRISPR/Cas9 parts for gene therapy of cancer. Zuris et al. modified 80% of their gene targets in human cells using a lipid-based nanocarrier that completed the effective delivery of Cas9/sgRNA RNPs (38). Currently, pH-sensitive nanocarriers have been used for a controlled release of CRISPR/Cas9 when internal of a tumor, which has a low pH of 5 to 6.5 in the tumor cells’ organelles and an extracellular environment, cytosol, blood, and healthy tissues with pH values of 7.4 (85). A variety of pH-sensitive components, such as copolymers, inorganic nanoparticles of crystals, lipids, and liposomes, have been formed for delivering CRISPR/Cas9 into tumors *via* a response to a change in pH. When the pH is lowered, for instance, the structural components of the nanocarrier would become degraded, and then the CRISPR/Cas9 cargo is released into the tumor (38). Nanocarriers built with glutathione (GSH)-sensitive potential can be used for CRISPR/Cas9 delivery because GSH is more concentrated inside the cell at 2 to 10 µM (86). Recently, these GSH-triggered nanocarriers have been formulated to deliver gene therapies, which include biologically reducible lipid nanoparticles (85), copolymers (34), and phenylboronic acid-derived lipid nanoparticles (69).

### Other nano-drug delivery systems for colorectal cancer

Porous nanoparticles are used more for delivering anticancer drugs because they have uniform pore sizes, have an organized physical shape, have alterable structures, and have larger surface areas (87). Porous nanoparticles as nanocarriers can produce an effective and controlled drug release that can be engineered by attaching stimuli-sensing pore-blockers or highly sensitive hybrid coats. Gold nanoparticles (AuNPs) have a large surface area. They can link with anionic drugs such as siRNA and plasmid DNA due to their having a cationic polymer surface *via* forming covalent bonds or an electrostatic interaction (88). AuNPs can increase cancer-targeting drug delivery by editing the cell-targeting components of the AuNPs, which can also improve passive permeation and retention effects (89). Applying a specific radiation wavelength can further spread the AuNPs for increased absorption and dispersion ability. Adjusting the AuNPs’ size, shape, and consistency can allow for effective biomedical diagnoses and examinations (90). Halal et al. used AuNPs to deliver cetuximab, and the AuNPs increased the endocytosis with significantly inhibiting downstream signaling pathways. Cell proliferation was reduced and the cell apoptosis amplified. However, many of these engineered nanoparticles may cause pulmonary inflammation by altering the membrane permeability, which may affect the particles ability to become dispersed beyond the lungs. A few of these chemically altered NPs may cause cardiovascular disease by impairing many vascular functions (91, 92). Gold nanoparticles (AuNPs) and carbon-based NPs can transit from the nose to the brain (91, 92). All chemically engineered NPs cause pulmonary inflammation (91, 92). Coated NPs can circulate systematically when inhaled. NPs can cause increased oxidative stress through inflammation and produce surface radicals (91, 92). NPs can cause the aggregation and increased accumulation of platelets, which lead to issues of blood clotting (91, 92). Additionally, because of the many and varied obstacles present *in vivo*, developing a nanocarrier for safely and efficiently delivering CRISPR/Cas9-based therapeutics continues to present many challenges.

### Challenges of CRISPR delivery

The first challenge requires efficiently encapsulating the CRISPR/Cas9 complex. There are three main techniques for applying the CRISPR/Cas9 for gene editing, which include using a plasmid encoding the Cas9 protein and the sgRNA, utilizing Cas9 mRNA with a sgRNA mixture, and through editing, genes using the complete Cas9 protein with the sgRNA ribonucleoprotein (RNP) (55). It is challenging to simplify and package a large Cas9 protein of 160 kD, an RNP size of 10 nm, and a mainly negative charge of the sgRNA surface into a single carrier for delivery (22). Additionally, after administration, these delivery systems still need to circumvent many physiological barriers *in vivo*, with each barrier and obstacle affecting the therapeutic outcome of the tumor treatment (22). CRISPR/Cas9 delivered through nanocarriers by being loaded into nanoparticles must pass the blood barrier, interact with degrading the enzymes of the plasma (93), overcome potentially being cleared by the phagocytes and macrophages (94), bypass the opsonization limiting the dispersion of the nanoparticles, and prevent the filtration of nanocarriers in the glomerulus (95). When the CRISPR/Cas9 loaded nanocarriers enter blood circulation, the nanocarriers must permeate through the barrier of the tumor tissue and increase in concentration before interacting with the target cells. Expanded vascular endothelial cells will also present many physical barriers when the nanocarriers traverse the tumor tissue.

The blood vessels in tumors leak and cause the expulsion of delivery systems from blood vessels. Since the extracellular matrix has a negative charge, the positively charged nanocarriers become easily compacted in the interstitial region (96, 97). It is difficult for nanocarriers to enter the tumor because it has limited lymphatic drainage, high interstitial pressure, is highly acidic and hypoxic and has a highly dense extracellular stroma. A transcellular obstacle in the membrane acts as a barrier for the CRISPR/Cas9 delivery system from the tissue of the tumor into the cell. After the nanocarriers with the CRISPR/Cas9 disperse from the endosomes or the lysosomes, they must enter the nucleus to begin the process of gene editing (38). These intracellular blockades include the cell membrane, endosome, and nuclear membrane. For CRISPR to become a highly effective therapeutic, it is essential to bypass all these intracellular barriers (38). Consequently, designing many delivery vectors requires optimization and improvement for an efficient CRISPR/Cas9 delivery, which is significant for using CRISPR/Cas9 as a therapeutic (38).

### Plant-derived exosome-like nanoparticles

Extracellular vesicles (EVs), such as exosomes, are nanoscale membrane-enclosed particles that help to orient the transport of proteins and genetic components (96–99). Exosomes can traverse the distance between cells, carry their cargo through the cell membrane, and deliver their contents that are biologically active (100). Since exosomes provide efficient and safe delivery of biomolecules, they have become increasingly attractive recently (101–103). Mammalian EVs have delivered siRNAs, miRNAs, drugs, proteins, and CRISPR/Cas9 molecules to formulate novel treatments and therapeutics (40). However, there are many limitations to the use of human exosomes as drug delivery carriers (102). One challenge includes fewer human exosomes that can be produced *in vitro* or collected from biological fluids. The production yield of exosomes affects the final cost of production and their practical applications in clinics (102).

Natural vesicles in plant cells can deliver agents and solve the present issues with existing nano-based delivery systems of therapeutics (102). PENs can be efficiently and frugally isolated from affordable edible plants in large quantities without toxicity. PENs innately carry different types of biomolecules, such as non-coding RNAs, and as a result, PENs can retain their stability when carrying their cargo from cell to cell (102). PENs are studied less than mammalian vesicles (102). Plant-derived exosome-like nanoparticles (PENS) are attracting increased attention to examine their application in disease therapy. Wang et al. were the first to develop PENs as nanovectors to deliver therapeutic drugs to brain tumors (104). They found evidence for the PENs’ ability to aggregate at specific tissues *in vivo*, in which the PENs could circulate long-term in the peripheral blood due to the high stability of the PENs. Researchers also discovered that plants could produce large-scale quantities of PENs. Although researchers are studying PENs at a higher rate, many previous studies describe the biogenesis and function of PENs. Early studies confirm plants’ production of exosome vesicles as a response to multiple biotic and abiotic stressors in the environment, which include pathogens and potential degradation from attack (105).

There is a need to improve the use of novel drug development strategies to treat diseases. Nanotechnology in drug development has become a promising approach; therefore, PEN-based therapies could become a new and novel strategy to treat cancers, inflammations, and immunological diseases. PENs are natural and innate nanoparticles released by edible plants such as grapes (106), grapefruit (107, 108) ginger, lemon (109), carrot (110), and many other plants. PENs provide plant chemical compounds that have many physiological and pathological activities. For example, ginger-derived exosomes (GDENs) decrease tumor cell proliferation, treat inflammatory bowel diseases and restore balance to the microbiota after the damage of tissues by monitoring gut bacteria (111–113). PENs are mostly non-toxic and do not cause inflammation. PENs are also safer than synthetic and artificial nanocarriers, including polymer-based nanoparticles, metal-based nanoparticles (gold or silver), and carbon-based nanoparticles (114–116).

### Lipid extraction and PEN reassembly

**Figure 3:**
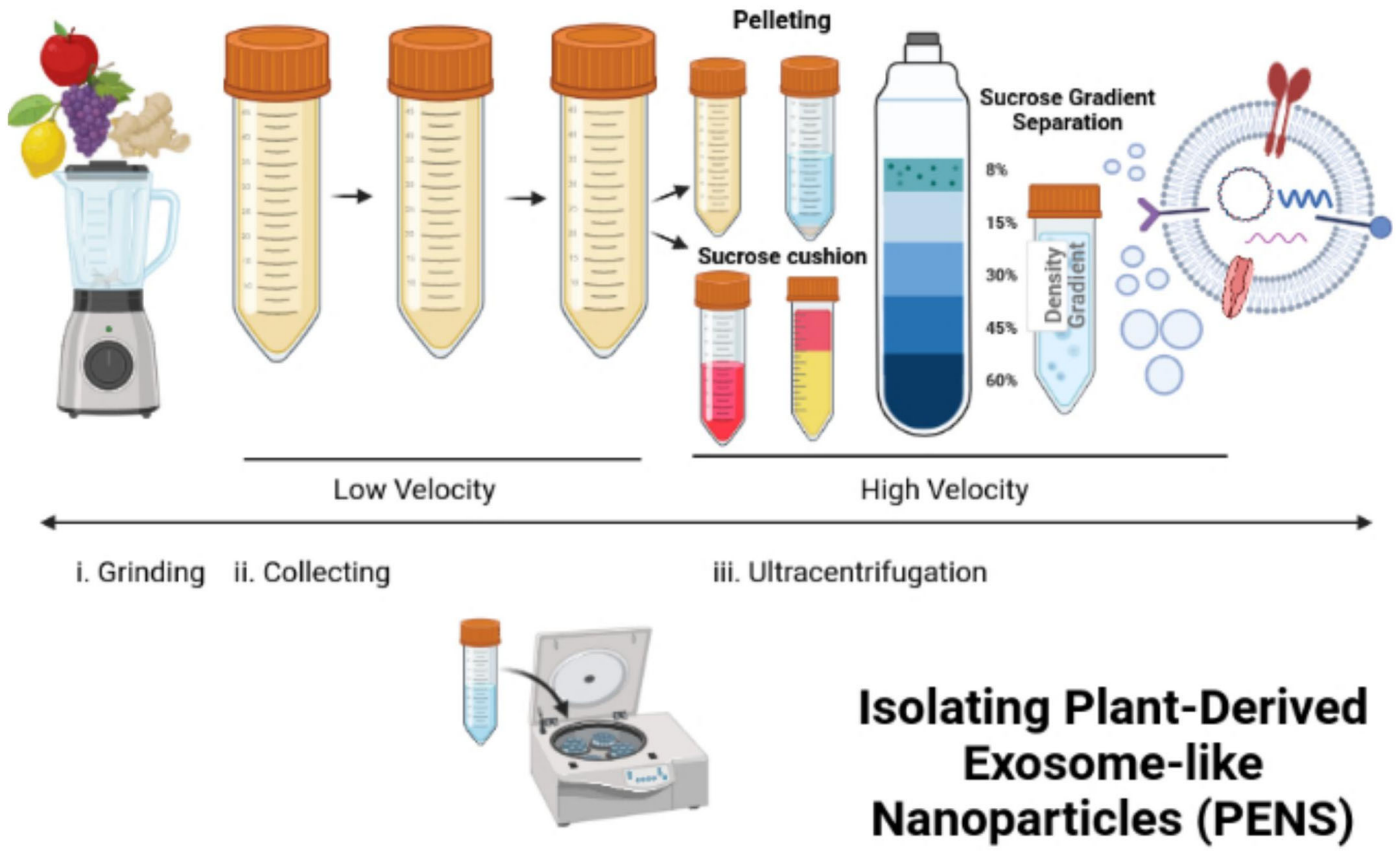
Isolation and reassembly of PENs. The figure presents the process of isolating plant-derived exosome-like nanoparticles (PENs) from fruits, such as apples, grapes, lemons, and ginger through low to high velocity ultracentrifugation and use of a sucrose gradient. Figure was retrieved from Kim et al. (22).

Differential ultracentrifugation can isolate PENs from plants (117–119). In this process, freshly squeezed plant juice becomes centrifuged at a low speed of 8,000 to 10,000 x g (**Figure 3**). The supernatant is centrifuged at a higher speed of 150,000 x g for 1 hour or more to collect a pellet of nano-sized particles (**Figure 3**). Then, the pellet dissolved in phosphate buffer solution (PBS) enters homogenization, and the added sucrose produces a gradient of 8%, 15%, 30%, 45%, and then60% (**Figure 3**). The remaining solution enters centrifugation at a rate higher than 150,000 x g for 2 hours (**Figure 3**). Different types of nanoparticles can be isolated based on the g-force of sedimentation and the varied gradients of sucrose (**Figure 3**). The best source of PENs is the 30% to 45% sucrose layer (117, 120, 121). About 100 grams of edible plants can produce 350 mg to 450 mg of nanoparticles (117, 119). A limitation in producing PENs include that it is challenging to produce them uniformly. This is challenging because natural PENs have many sizes, from 50 nm to 500 nm (117, 119, 120, 122, 123). Many research studies report that PEN lipids should be isolated and assembled into a more uniform-sized nanoparticle before or after cargo loading (117, 124, 125).

The two main types of lipids in PENs are phospholipids and glycerol lipids. These two lipids can gather in the organic phase during the two-phase liquid-liquid extraction (LLE). Zhang et al. (126) utilized the Bligh and Dyer method (127, 128), an LLE method, to isolate lipids from ginger nanoparticles and load siRNA-CD98 into the lipid/HEPES buffer solution. A 200-nm liposome extruder transfers the extracted lipids. An effective uniform-sized siRNA-CD98/ginger-derived lipid nanovesicle consists of an average diameter of 189.5 nm of size. The delivery of the nanoparticles to the colons of mice was efficient and successfully treated their ulcerative colitis. Wang et al. used the identical Bligh and Dyer procedure and extracted total lipids from grapefruit (117). After Wang et al. (2013) sonicated the lipids and passed them through a homogenizer, the lipids formed into flower-like nanoparticles of 200 nm in size.

### Loading of cargo into PENs

Small molecular drugs, siRNAs, and DNA expression vectors can be loaded into PENs to help target many disease tissues (117, 118, 124, 126). Because PENs have a negative charge, positively charged compounds such as drugs can be quickly loaded into the PENs *via* sonication (117, 124). Neutral and negatively charged molecules loaded into PENs display no adverse effects on their biological functions (117, 118). Because PENs are highly hydrophobic, they can override the static electronic forces at the surface level, which guides them in the process of bioencapsulation.

### PENS compared to other nanotechnologies combined with CRISPR therapy

The editing efficiency of lipid nanoparticles (LNPs) that carry CRISPR/Cas9 plasmids is low because their delivery efficiency into cells has been poor during *in vivo* experiments with animals (129). Thus, the LNP delivery of CRISPR/Cas9 plasmids has not fulfilled the clinical requirements due to their reduced editing and delivery efficiencies (129). Currently, it is challenging to deliver RNPs for targeting organs in the body because it is arduous to develop stable nanoparticles for systemic administration and delivery (129). Many researchers have established a universal procedure for engineering stable RNPs by adding cationic elements to ionizable LNP products. In these processes, the lipids guide theencapsulation of the RNPs by maintaining the activity and directing the DNA editing in the target tissues. LNPs have an 80% gene editing efficiency with a 70% editing efficiency for amino ionizable nanoparticles *in vitro* and *in vivo* (130). Polymeric nanoparticles (PNPs) such as Poly(lactic-co-glycolic acid) (PLGA) have an editing efficiency of 95%, and polyethylenimine (PEI)-β-cyclodextrin cationic polymers for nanoparticles edit at 19.1% *in vitro* (130). INPs edit at an efficiency of 60% *in vivo* and *in vitro*. NPs formed with DNA nanowires have an editing efficiency of 36% *in vitro* and *in vivo* (130). Gold Nanoparticles combined with lipids (lipid/AuNPs), forming complexes, were used to encapsulate the CRISPR-Cas9 system and then thermally stimulated to release the contents (131). The lipid/AuNPs encapsulated the CRISPR-Cas9 DNA plasmids at an efficiency of 97% with a release efficiency of 79.4% when triggered by irradiation from a pulse laser (131). The lipid/AuNP encapsulated CRISPR/Cas9 displayed high stability in the blood and exhibited more extended periods of circulation in the body (131).

Lipid nanoparticles can be formed with ionizable lipids that are biologically degradable, such as with PEG-DMG-lipid nanoparticles encapsulating Spy Cas9 mRNA and the sgRNA. These ionizable lipids can deliver the CRISPR/Cas9 units *in vivo* to edit genes, yielding a retained gene knockout for 52 weeks after a one-time administration (129). The LNP encapsulation of the CRISPR/Cas9 components reduced the protein expression of TTR in mice by 97% or more (129). Lipid nanoparticles should be further considered as effective delivery systems for gene therapy. Zwitterion amino lipids were formulated and delivered long RNAs of Cas9 mRNA and sgRNA *in vitro* and *in vivo*. The Zwitterion amino lipids maintained a 95% reduction in protein (129). The α-helical cationicpolyamine acid PPABLG was produced and displayed an increased potential to permeate through the cell membrane and yielded a high efficiency of endosomal escape. When combining CRISPR/sgRNA plasmids with copolymers, 60% of the Cas9 was expressed, and 35% of the polo-like kinase gene was knocked out (129).

DNA nano complexes are a new delivery system with an efficient loading capacity, high biocompatibility, biodegradability, a high level of cell uptake, an improved endosomal escape, and high efficiency of genome editing. Arginine-gold nanoparticles (ArgNPs) can deliver chemically altered Cas9 proteins with the targeting sgRNA, which resulted in a high delivery efficiency into the cytoplasm and the nucleus at approximately 90% (129). ArgNPs sustained genome editing with an efficiency of 23 to 30%. Most off-target effects were decreased (129). Poly(lactic-co-glycolic acid) (PLGA) nanoparticles were used as nanocarriers engineered to deliver a CRISPR/Cas9 plasmid into macrophages produced from bone marrow (132). These PLGA-nanocarriers had a size of 160 nm and encapsulated fluorophore 6,13-bis(triisopropylsilyl ethynyl) pentacene (TIPS pentacene). A PLGA nanocarrier capped with an amine group encapsulated about 1.6 wt% of DNA at an encapsulation efficiency of 80% (132). Most of the DNA was released within 24 hours, with 2 to 3 plasmid copies released from each nanoparticle (132).

In comparison, PENs have innate and specific-cell-targeting capacities. However, the mechanisms that underlie the targeting abilities of PENS are lacking and limited since PENS have not been as extensively studied as mammalian derived exosomes (MDE)s (22). The uptake of PENs by certain cells has been linked to genes encoding miRNA and siRNAs with small molecules localized in and on PENs that become extracellular ligands for the targeting components of the cells. PENs have high intrinsic and innate targeting potential, lowered off-targeting effects, and no identifiable toxicity compared to artificial nanoparticles (22). For these reasons, PENs do not require additional modifications to enhance their biocompatibility, *in vivo* adherence and stability, and pharmacokinetic properties. PENs have an assurance of high biocompatibility and stability when under many different physiological conditions, such as in the bloodstream and at different pH levels (22). PENs can function as vectors to be encapsulated with siRNAs and miRNAs, chemotherapeutic drugs, and hydrophobic compounds. PENs can circulate in the blood for long periods of time, which provides potential therapies for tumors and other chronic diseases (78).

There are many different methods of loading cargo into PENs. The passive loading procedure involving co-incubating exosomes and drugs allows PENs to become loaded with cargo molecules (22). Contrarily, passive loading procedures may not achieve an increased yield of encapsulation. Sonication, freeze-thaw cycling, and other manual processes can temporarily disrupt the cohesion of PEN membranes and improve cargo loading efficiency. To evade the potential of toxicity *in vivo*, which may be a cause of impurities, and to acquire nanocarriers of uniform sizes, nano vectors can be formed by using extracted lipids from PENs and then mixing the cargos with the extracted lipids while preparing the lipid thin film (22). This method can load more cargo, such as siRNA, antibodies, DNA expression vectors, and lipophilic drugs (22). For lipophilic drugs, such as doxorubicin, these encapsulation methods with PENs have provided high encapsulation rates of 95.9% ± 0.26%, yet the encapsulation efficiency of many other cargoes have not been found or explored (22). PENs in the colon

Ju et al. confirmed that grape-exosome-like nanoparticles induce the restoration of intestinal stem cells *via* the Wnt/β-catenin signaling pathway, which controls the genes of AXIN-2, Cyclin D1, c-MYC, and EGF (106). The grape-derived exosome-like nanoparticles (GELNs) showed a high cellular uptake by intestinal stem cells, in which a clathrin-mediated endocytosis inhibitor did not impact their uptake (106). It is presumed and accepted that there are specific ligands and routes of receptors between PENs and intestinal stem cells. Yet, it has been challenging to determine the functions of particular chemical molecules and ligands in PENs since the mechanisms of delivery and the internalization of PENs into cells still lack clarity. Although PENs are similar to mammalian-derived exosomes (MDEs), PENs differ from MDEs in many aspects. MDEs have lipid bilayers that consist of cholesterol, glycosphingolipids, ceramides, and phosphatidylserine, which give stability and a specific rigidity (133, 134). Contrastingly, the membranes of PENs contain phosphatidic acid (PA), phosphatidylcholines (PC), di galactosyl diacylglycerol (DGDG), and monogalactosyldiacylglycerol (MGDG) (135), in which these lipids provide intrinsic mammalian-cell-regulatory functions.

PA is the most well-known of the phospholipids for its potential to target and stimulate the mammalian target called rapamycin (mTOR), commonly identified in PENs. The mTOR pathway regulates cell growth, proliferation, and restoration of functions in many human health and disease processes. PC is a resource of choline in the body that may prevent damage to a cell wall in the large intestines by establishing cellular blockages in the cell membrane. Teng et al. purified GDENS and examined their genes, lipids, and proteins (135). Teng et al. discovered that phospholipid-enriched membranes of GDENS allowed their advantageous uptake by the microbiota that could help monitor the gut bacterial microbiome (135). Through the proteins and genes present in the GDENs compositions, the GDENS could assist with adjusting the intestinal microenvironment. To address the pharmacodynamics of PENs, because PENs are generated naturally by plants, give stability and rigidity, and have a well-suited morphology, they can encapsulate drugs within their lipid bilayer and target the tissues sought for treatment (135). PENs are membranous vesicles with site-specific targeting (136). These qualities of PENs, having a particular organization of proteins and lipids, allow them to alter genes for therapy, transfer drugs, prevent an immune response, and classify PEN as highly beneficial for future applications in medicine and treatment.

For the pharmacokinetics of PENs, they can safely transfer drugs and circulate in the blood for long periods after a systemic administration, offering a promising targeted delivery vehicle for disorders that cause tumors and other chronic diseases (136). To analyze the ability of PENs to cause toxicity, Zhang et al. used ginger-derived nanovectors (GDNVs) to treat tumors and evaluated whether the GDNVs could damage any tissues or organs (111). For the pharmacodynamics of PENs because the PENs were targeted entirely to the tumors, there was a reduction in the accumulation of GDNVs in the spleen and the liver, which lessened systemic toxicity from the drugs with a prolongation of the drugs circulating in the blood. The histological tests of the spleen, liver, lung, kidney, or heart showed no pronounced degradation to these organs compared to their control sample groups, which confirms that PENs can be used as a drug delivery nano-system, improving drug efficacy and lessening the toxicity of drugs (22). Zhang et al. (111) showed that GDNVs loaded with Doxorubicin (Dox) released their cargo in an acidic-like tumor microenvironment, and the GDNVs allowed an increased diffusion of the Dox more than the commonly used commercial liposomes. PENs can deliver chemical drugs, small molecules, and genes because they have high biocompatibility with immense biodistribution potential. As a consequence, PENs can deliver drugs with an accurate and specific targeting of tissues without causing systemic effects, causing an optimized therapeutic effect and lesser negative side effects (22).

Drug delivery systems (DDS) that carry siRNA and miRNA have low loading efficiencies with their less effective therapeutic effects, causing some adverse effects (137). To address these issues associated with GDENS, Zhang et al. extracted the lipids from the GDENS and then loaded them with the siRNA to target CD98 for treating ulcerative colitis (22). For the mechanism of action for PENs, Zhang et al. produced GDENs that had high biocompatibility without substantial toxicity, causing more apoptosis of macrophage and colon-26 cells *in vitro* than commercially used DC-Chol/DOPE liposomes (22). GDENs were transfected with siRNA to target CD98 using sonication and then applied to examine the mRNA expression of CD98 in an *in vivo* test. These siRNA-CD98-loaded GDENS were maintained in the gastrointestinal tract after oral administration and immensely lessened CD98 expression in the intestine compared with randomly siRNA-loaded GDENs. Ginger-derived lipid vectors (GDLVs) were discovered to efficiently carry divalent metal transporter 1 (DMT1)-siRNA blunts to intestinal epithelial cells to mediate the process of loading iron in the inherited hemochromatosis (136). To optimize the GDLV’s effectiveness in targeting the duodenum, the GDLVs were fused with folic acid (FA), allowing it to be interspersed into the duodenum and jejunum through the proton-coupled folate transporter. The siRNA-FA-GDLVs were given to mice, and the iron loading was diminished by a reduction of the Dmt1-mRNA expression, causing decreased levels of ferritin, TSAT, and non-heme Fe in many organs, which included the kidney, heart, pancreas, and the liver.

### An oral administration of PENs

Oral administration is the more selected and endorsed drug delivery route since it is the most facile and convenient for pharmaceuticals. An oral administration offers a low risk of infection, which differs from direct injective routes, enhances the permeability of the entire gastrointestinal tract, and can evade blood clearance (138). Oral administration is non-invasive and has been used for delivering MDEs. Lin et al. delivered bovine and porcine-milk-derived exosomes that contained miRNA through an oral administration, and they detected the bovine and porcine-milk-derived exosomes in the intestinal cells (139). There have not been many MDEs developed for oral administration (140). A majority of MDEs were not designed for oral delivery because of their low stability at many different pH levels and temperatures, their quick degradation in the digestive tract, and their limited ability to be produced at an industrial rate for oral dosing (141, 142). PENs have been developed for oral administration. PENs can be administered in many different methods because they have high tolerability, the potential to target specific tissues, and high biocompatibility. As a consequence, an oral administration of PENs can beget many rapid effects in pharmacotherapy (136). For example, grapefruit-derived nanovectors (GNVs) carried MTX to intestinal macrophages after an oral administration (22), in which these GNVs targeted the intestinal macrophages with greater efficiency than most common and commercial liposomes.

Researchers placed PENs in different pH levels of water, O.5 N of NaOH, and O.5 N HCl to examine the stability of PENS. The PENs were confirmed to have a reduced size when in acidic solutions (22), the PENs in alkaline solutions have no effect. PENs were then placed in solutions with gastric and intestinal enzymes to analyze the stability of PENs during digestion. The GNVs were increasingly repellent to digestion by gastric pepsin, the intestinal enzyme of pancreatin, and resistant to the bile solutions (22). The intestinal-like fluids had no effect on PENs derived from grapefruit, carrots, and grapes; however, the GDENs decreased the negative charges (110). In acidic conditions, PENs display a reduction in surface charge. For example, the surface charge on GDENs altered from negative to positive in the stomach acidic-like fluids and then returned to having the negative surface charge in the intestine-like solutions (143). For the proof of concept, after an oral administration of PENs delivering CRISPR/Cas9 components for targeting and treating CRC, they can be absorbed by target cells or tissues. For example, GDENs primarily accumulate in the liver after an oral administration, with none identified in the lungs, spleen, or other organs (143). This interesting propensity of PENs to be more concentrated in the liver may reduce the negative effects of anti-cancer drugs, which have a non-specific dispersion of therapeutics. GDENs were taken up by albumin plus hepatocytes; however, grapefruit-derived exosomes like nanovesicles were accumulated more in F4/80+liver Kupffer cells, showing that PENs that are derived from different plants have dissimilar and distinctive targeting capabilities. These findings also confirm the hypothesis that GDENs transit to the liver from the gut through vascular vessels (143).

PENs can deliver hydrophobic anticancer drugs and genes to target cells or tissues *via* oral administration. An anti-inflammatory drug termed MTX was loaded into grapefruit-derived exosome-like nanovesicles that targeted F4/80+ macrophages in the lamina propria and kept the MTX therapeutic effects. PENs transfected intestinal macrophages more efficiently than commercial liposomes after an oral administration. GDENs deliver RNA to target bacterial genes (135). The gut bacteria selectively interacted with and engaged the GDENs. After an oral administration of GDENs, genes were delivered into the intestines of mice, which were analyzed by qPCR tests of GDENs miRNA in the gut and feces after the treatment. GDENs also abated mouse colitis by impacting the distribution of the gut microbiota. Thus, PENs are favored as imperative candidates for oral-delivery materials. Many of the PENs discussed have been delivered through an oral administration, as most MDEs or liposomes have been commonly delivered by IV injection. An oral administration of PENs ensures the therapeutic effect is maintained with ameliorated targeting capability; however, PENs also guarantee a decreased risk of infection, whereby the therapeutic methods of using PENs *via* oral administration could be an excellent procedure to enhance patient participation and compliance in clinical trials.

In addition, plant-derived extracellular vesicles (PEV) can be extracted from plants, such as grapes, grapefruits, other fruits, vegetables, and spices (144). PEVs isolated from grapes and grapefruits are highly therapeutic (144). Ginger has a high efficiency in drug delivery with a substantial therapeutic ability (144). There are a few doubts about using natural plant vesicles as drug carriers because they can aggregate during purification and have a low cargo loading capacity. Since PEVs may aggregate during ultracentrifugation, PEVs are ineffective after intravenous administration. By adding multiple purification procedures when isolating the PEVs or reconstructing the plant nanovectors and then assembling the molecules that consist of their membranes, PEVs can become more efficacious (118). Nanovectors have been formed from grapefruit-derived lipid nanoparticles and utilized as a delivery system for chemotherapeutic drugs and siRNAs (145).

In a study by Garaeva et al. (102), the researchers used *in vitro* models to show the significant and efficient uptake of the fluorescently labeled proteins of HSP70-Af647 or BSA-AF647 packed into grape-fruit extracellular vesicles (GF-EVs) by human cells versus free proteins. Garaeva et al. (102) observed that the grapefruit vesicles were highly efficient when carrying the exogenous proteins into the human cells *in vitro*. For their *in vivo* analysis of the vesicles’ ability to distribute into the cells, they injected the 125I-BSA-loaded GF-EVs into mice intravenously. Garaeva et al. (102) found that loading the GF-EVs with the labeled protein did not alter the surface of the vesicles. The *in vivo* results showed that the animal tissues had a substantially effective uptake of the protein-loaded GF-EVs. The biodistribution patterns for the GF-EVs were similar to the human exosomes in mice (103). The results of the Garaeva et al. study succinctly demonstrated the high efficiency of native GF-EVs to deliver exogenous proteins into mammalian cells and tissues safely. The results from the Garaeva study and many preceding studies (118, 124, 126, 145, 146) establish a foundation and basis for further studies and the development of similar plant vesicle delivery systems for their application in novel therapeutics and medicine.

**Figure 4:**
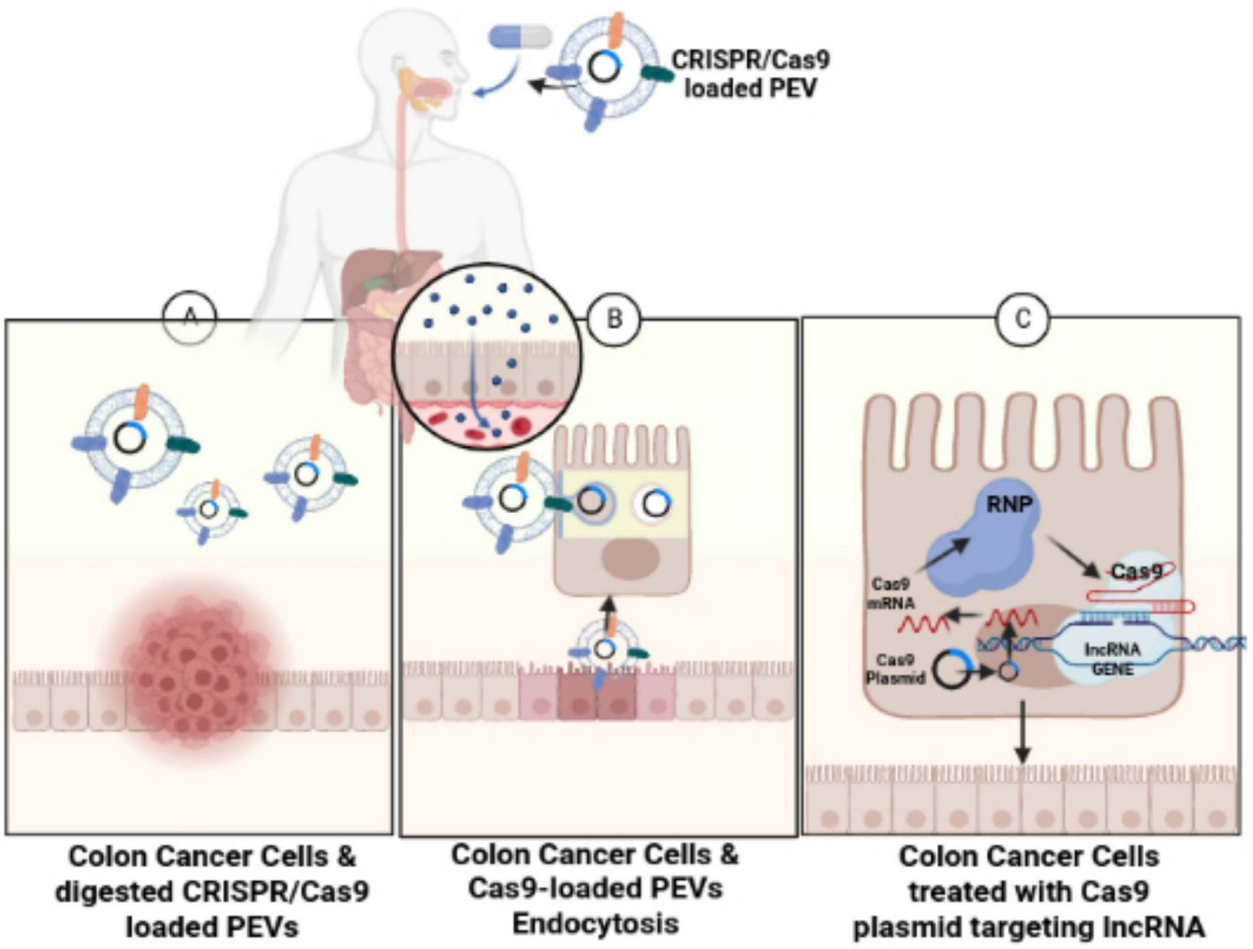
Treating colon cancer cells with CRISPR/Cas9-loaded PEVs. The figure conveys schematic representation for the oral administration of CRISPR/Cas9-loaded PEVs to the target lncRNAs present in colon cancer cells. **(A)** Figure shows the digestion of the CRISPR/Cas9-loaded PEVs into the colon. **(B)** The CRISPR/Cas9-loaded PEVs progress through endocytosis into the intestinal epithelial cells. **(C)** The Cas9-targeted treatment of lncRNA in colon cancer cells eliminates the cancerous colonocytes.

In slight contrast, this review and study suggest an oral administration of CRISPR/Cas9 plasmid DNA-loaded PEVs similar to the protein-loaded GF-EVs used in the Garaeva et al. study, rather than an intravenous administration, for the treatment of colon cancer cells (**Figure 4**). The CRISPR/Cas9 plasmid DNA-loaded PEVs could specifically target lncRNAs, such as LINC01296, to reduce the proliferation of SW480 and SW620 colon cancer cells. An oral administration of CRISPR/Cas9 plasmid DNA-loaded PEVs is recommended instead of an intravenous route because the CRISPR/Cas9 plasmid DNA-loaded PEVs can traverse through the digestive tract directly into the large intestines or the colon. PENs have a negative transmembrane potential on their surface from -12 mV to -17.1 mV (117). In the pH of the intestines, the PENs from grapes, grapefruit, and carrots have no change in the net charge on their surfaces. However, when these PENs enter the acidic pH of the stomach, the PENs’ negative charges are significantly reduced since the acidic conditions neutralize the negative charges (117).

PENs are non-toxic and do not create a significant immune response, mainly because of their extraction from edible plants. Within 12 hours, 20% of B cells and 14% of T cells uptake the PENs, such as grapefruit-nanoparticles (GNPs), after treatment (117). The uptake of the GNPs also increased at a higher temperature. Wang et al. found grapefruit PENs are more stable than positively charged liposomes at 37°C in 10% bovine serum (117). The GNPs were stable at room temperature for more than a month, during which its loaded cargo was still bioactive during the month (117). Zhang et al. discovered that PENs from ginger-derived nanoparticles were increasingly more stable in stomach-like and intestine-like conditions and could withstand freeze with thaw cycles (117). Because PENs such as GNPs are more resistant to extreme temperatures and can endure stomach acids, PENs can deliver CRISPR-based gene therapies through and into the GI tract. An oral administration of CRISPR/Cas9 plasmid-DNA-loaded PEVs may ensure its direct delivery into the large intestines for treating colon cancer. Nevertheless, more research studies are required to confirm the safety and complete efficiency of this specific type of plant-based nanocarrier for application as a CRISPR/Cas9 oral delivery system.

Additionally, there are limitations for an oral administration of CRISPR/Cas9 DNA-loaded PEVs. In a neutral pH, the diameters of the plant-derived nanoparticles from ginger, grapefruit, grapes, and carrots are all lower than 300 nm (117). However, in an acidic pH, such as the pH of the acidic stomach, the size of the grape-derived nanoparticles dwindles, ginger-derived nanoparticles size increases, the grapefruit-derived nanoparticles divide into different sizes, and the two sizes of the carrot-derived nanoparticles become conjoined together in a single subset, which is larger (117). The sizes of these nanostructures vary since they alter their size in different solvents, with different buffer concentrations, and in extreme temperatures. Therefore, the plasmid-DNA encoding the CRISPR-Cas9 components should be loaded into the PEVs, since the plasmid-DNA of the CRISPR/Cas9 system will better fit into the PEVS that have sizes that can become altered by the acidic pH of the stomach. Another limitation is that siRNAs and DNAs have PEV loading efficiencies lower than positively charged therapeutics (109, 117). Sonication with freeze-thaw cycling may briefly deconstruct the PEN membranes and optimize the loading efficiency of the cargo. To prevent possible toxicity *in vivo* and to attain nanocarriers of uniform sizes, the formation of nanovectors can incorporate the lipids from PENs. Then the cargo is added when preparing the thin film of lipids (146). Utilizing this strategy of cargo loading can be effective for loading the lipophilic drug of doxorubicin, biomacromolecules such as siRNAs, antibodies, and DNA expression vectors (108, 145).

## Discussion

PENs can offer tremendous therapeutic advantages compared to mammalian-derived exosomes (MDEs) or synthetic nanoparticles. The benefits of PENs include a less complicated method of mass production (113), less toxicity, decreased immunogenicity (147), an efficient uptake by cells (145), and high biocompatibility with increased stability (148). Many studies have confirmed the efficacy of PENs; however, many of the properties and bioactivities of PENs are not presently and wholly understood. Additional research studies are needed to improve our understanding of the functions and application of PENs.

Many previous studies report that PENs have similar properties to MDEs (149), in which PENs have been used for treating various diseases (150, 151). PENs can be acquired from large-scale manufacturing methods from many renewable sources (152), which can meet the demand for urgent production of high-quality exosomes. The natural components of PENs allow enhanced biocompatibility and increased safety with less cytotoxicity, and PENs also have reduced negative side effects. There are also many sources of PENs available, which many researchers can select from this diverse pool of nanovesicles depending on their applicability and potential to treat a disease. PENs have similar innate therapeutic materials and components to MDEs, which can be transferred and attached to the targeted cells. PENs can be used as nanocarriers because their lipid membranes are increasingly stable and can be easily altered to target specific ligands. Additionally, PENs can be rapidly examined by using eco-friendly procedures (153). The standardized process for producing PENs can become founded and organized by categorizing and characterizing PEN nanovesicle variations.

However, there are a few disadvantages of PENs. The main disadvantages include that PENs are heterogeneous in size and in physicality, which PENs may be considered and recognized as impurities by the body, which may cause unfavorable immune responses with other activities and mechanisms of regulation that have not been studied during treatment (154). The activities and roles of PENs are still not fully understood and lack much clarity; therefore, effects that have not yet been predicted can occur with the recognition of an unidentified biological material. During the application of the PENs, a few challenges to biosafety and toxicity can result from the unknown bioactive components of the plants. Because PENs are not developed from bodily fluids, tissues, or cells (155), PENs may have a reduction in targeting potential in specific tissues in the body. For future research studies to reduce and surmount these disadvantages of using PENs, their isolation processes should be enhanced to form more uniform nanovesicles. An evaluation of the morphologies, quantities, and chemical consistencies of PENs should be obtained to identify their functional roles and properties.

PENs have been tested in preclinical and clinical trials because they can transport cell-generated contents and are well-suited for industrial large-scale productive yields by standardized manufacturing processes (22). PENs are more underdeveloped than MDEs. PENs derived from grapes (NCT01668849), ginger, and aloe (NCT03493984) have been chosen and registered for clinical trials. Miller et al. affixed curcumin with plant exosomes and applied this treatment to participants with colon cancer (NCT01294072); however, this study has not initiated the recruitment phase (22). Although PENs are highly advantageous, their production processes during the stages of cultivation, isolation, and classification into clinical trials and manufacturing have not been completely executed (22). To subjugate these challenges in the clinical applications of PENs, the problems in good manufacturing practice (GMP) production of PENS should be considered, and GMP-quality grade PENs should be further examined and inspected.

This study’s main focus and purpose was to describe PENs as effective delivery vehicles of CRISPR/Cas9-based therapeutics for CRC. This review described the CRISPR/Cas9 system, targeting lnRNAs of colon cancer cells with CRISPR/Cas9 technology, discussed the challenges of the current delivery systems for CRISPR-based therapeutics, and provided established evidence for the use of PENs as nanocarriers to more efficiently deliver CRISPR-based therapeutics. This study examined the potential use of PENs and PEVs for delivering CRISPR-based therapeutics more directly into colon cancer cells *via* an oral administration versus the standard intravenous route. The intravenous route presents many barriers for nanocarriers to overcome for a final arrival into the digestive tract and the colon. The use of the oral route of administration also presents many obstacles and barriers, such as the stomach’s high acidity and interactions with the bile salts of the intestines. However, an oral administration may serve as an effective and more direct delivery of CRISPR-Cas9-loaded PEVs through the digestive tract into the colon for potentially treating rectal colon cancer. Because PEVs can resist degradation from stomach acids and high body temperatures, PEVs can successfully traverse through the digestive tract into the colon. Targeting the lncRNAs of colon cancer cells with CRISPR/Cas9-based therapeutics may become more efficient using PEVs as nanocarriers administered *via* an oral route through the digestive tract. The oral route may provide a plain and straightway delivery of CRISPR/Cas9 loaded-PEVs into the colon to eliminate colon cancer cells.

Research studies on the oral administration of CRISPR-Cas9-loaded PEVs are immensely needed. Therefore, this study is limited in describing the complete effects of CRISPR-cargo loaded PEVs processed through the digestive tract. Further research is needed to confirm the complete safety and efficiency of PEVs as a digestive delivery system for CRISPR/Cas9-based therapeutics to treat colon cancer cells. Future research studies could examine any safety concerns, and the successful delivery of CRISPR/Cas9 loaded PEVs into the colon *via* an oral route to more directly target colon cancer cells. This review may represent one of the many earlier studies of plant-derived exosome-like nanocarriers that may assist with furthering the investigation of PENS and PEVs as novel nanocarriers of CRISPR-based therapeutics.

## Author contributions

The author confirms being the sole contributor of this work and has approved it for publication.
